# A Highly Selective Electrochemical Sensor Based on Molecularly Imprinted Copolymer Functionalized with Arginine for the Detection of Chloramphenicol in Honey

**DOI:** 10.3390/bios13050505

**Published:** 2023-04-28

**Authors:** Tingrun Lai, Hui Shu, Bo Yao, Siying Lai, Ting Chen, Xuechun Xiao, Yude Wang

**Affiliations:** 1 National Center for International Research on Photoelectric and Energy Materials, School of Materials and Energy, Yunnan University, Kunming 650504, China; 2 Institute of Materials Science and Devices, School of Materials Science and Engineering, Suzhou University of Science and Technology, Suzhou 215009, China; 3 Yunnan Key Laboratory of Carbon Neutrality and Green Low-Carbon Technologies, Yunnan University, Kunming 650504, China

**Keywords:** chloramphenicol, arginine, molecularly imprinted polymer, electrochemical sensor, honey

## Abstract

Developing an efficient method for chloramphenicol (CAP) detection is of great significance for food safety. Arginine (Arg) was selected as a functional monomer. Benefiting from its excellent electrochemical performance, which is different from traditional functional monomers, it can be combined with CAP to form a highly selective molecularly imprinted polymer (MIP) material. It overcomes the shortcoming of poor MIP sensitivity faced by traditional functional monomers, and achieves high sensitivity detection without compounding other nanomaterials, greatly reducing the preparation difficulty and cost investment of the sensor. The possible binding sites between CAP and Arg molecules were calculated by molecular electrostatic potential (MEP). A low-cost, non-modified MIP electrochemical sensor was developed for the high-performance detection of CAP. The prepared sensor has a wide linear range from 1 × 10^−12^ mol L^−1^ to 5 × 10^−4^ mol L^−1^, achieves a very low concentration CAP detection, and the detection limit is 1.36 × 10^−13^ mol L^−1^. It also exhibits excellent selectivity, anti-interference, repeatability, and reproducibility. The detection of CAP in actual honey samples was achieved, which has important practical value in food safety.

## 1. Environmental Implication

In this study, the whole process of material preparation, characterization, analysis, and performance testing does not produce toxic substances and has no impact on the environment.

## 2. Introduction

CAP is a kind of amide alcohol antibiotic, which is widely used in poultry disease prevention because of its good inhibitory effect on Gram-positive bacteria and Gram-negative bacteria [1,2]. However, excessive or irregular use of chloramphenicol will make it residual in animals or cause it to enter the food chain and the environment [3,4]. Due to the toxicity of chloramphenicol, over a long time, the intake of a large number of foods with excessive CAP residues will lead to its accumulation, which makes the human body produce drug resistance and cross-resistance to similar drugs. It can also cause gastrointestinal symptoms, abnormal liver function, abnormal blood system, and intestinal flora imbalance [5]. Based on the above adverse effects, China’s Ministry of Agriculture has announced that CAP has been included in the list of banned drugs for food animals [6,7]. However, due to the low price and effectiveness of CAP, there are still many food animal breeders who use it illegally [8,9]. Reports of unqualified CAP content in food sampling have also occurred. Therefore, it is important to find an efficient and accurate method to detect CAP content in food. At present, many methods have been applied to the quantitative detection of CAP, such as high-performance liquid chromatography-tandem mass spectrometry, gas chromatography, capillary electrophoresis, colorimetry, and so on [10,11,12,13]. Although these traditional detection methods have high accuracy, they also have the disadvantages of high cost and long time consuming [8,14]. Hence, it is especially crucial to find an alternative CAP detection method with high accuracy, low cost, and rapid speed.

The electrochemical sensor is a new CAP detection technology that can replace the traditional method due to its high sensitivity, simple operation, low cost, and rapid speed [15,16,17]. As an important part of electrochemical sensors, molecular recognition units play an irreplaceable role in the final detection performance. Antibody, aptamer, and enzyme are commonly used as molecular recognition units in the electrochemical sensors. However, these recognition units are susceptible to environmental factors such as temperature and pH values, resulting in inaccurate detection. Their sensitivity and selectivity also need to be improved [18].

The molecular imprinting technique, a new artificial molecular recognition method, has attracted the focus of many researchers in the field of electrochemical sensors owing to its simple preparation process, environmental factors’ anti-interference ability, and good selectivity [19,20,21,22]. It forms the polymer material through the covalent or non-covalent bonds between appropriate functional monomer and template molecules. We used the elution solution to remove the template molecule from the polymer, leaving a hole site with the same shape and structure as the template molecule. The probe ion in the electrolyte can be exposed to the electrode through this site and then generates an electric signal [23,24]. When the hole is combined with the analyte, it hinders the electron transfer and makes the electrical signal change, thus achieving the high selectivity quantitative detection of the analyte.

As an important part of the MIP formation process, the functional monomer affects the electrochemical performance of the final polymer. To date, many organic compounds have been used as functional monomers to detect CAP. Zhao [25] used diethyl aminoethyl methacrylate (DMA) as a functional monomer, composite carbon nanotubes, and gold nanoparticles. The final CAP detection linear range was 3.1 × 10^2^–3.1 × 10^5^ nM, and the minimum detection limit (LOD) is 74 nM. Roushani [2] employed hydroquinone as a functional monomer, composite silver nanoparticles, and 3-aminomethylpyridine functionalized graphene oxide. The final CAP detection linear range was 1 × 10^−3^–1 nM, LOD = 3 × 10^−4^ nM. Yang [5] used 3-hexadecyl-1-vinyl imidazolium chloride (C16VimCl) as functional monomer composite carbon nanotubes for CAP detection; linear detection range was 5–5 × 10^2^ nM and 5 × 10^2^–4 × 10^3^ nM, LOD = 0.1 nM. Although these MIP electrochemical sensors can achieve the quantitative detection of CAP, different nanomaterials are often needed in the preparation process to achieve a wide linear range and low detection limit, which increases the cost and complexity of CAP electrochemical sensor preparation. Thus, it is of great significance to find a new way to achieve high-performance detection of CAP without compounding other nanomaterials, thereby reducing the production cost and complexity of electrochemical sensors.

In recent years, many researchers have used conductive polymers to modify the electrode surface and improve its electrochemical performance. Arg, as a kind of low-cost, non-toxic, biologically inclusive, and highly electrocatalytic organic matter, has been modified on the electrode surface by electro-polymerization in many studies [26,27]. The surface obtained by electro-polymerization has good uniformity, high adhesion stability, and a large surface area. In addition, Arg, as an alkaline amino acid, usually exists in the form of zwitterionic. There are three nitrogen atoms in the guanidine group, and a positive charge is distributed, which can form hydrogen bonds with special characteristics. Its multidentate characteristics also enable Arg to produce long-chain hydrogen bonds and electrostatic behaviors with negative charge groups, thereby enhancing the electronic flow between the target and the functional electrode, thus improving the detection sensitivity [28,29].

In this study, Arg was selected as the functional monomer for MIP. A molecularly imprinted membrane with plenty of reactive sites was formed on the surface of glassy carbon electrode (GCE), which was no longer combined with other nanomaterials. This can not only reduce the production cost and complexity of the sensor, but also improve the sensitivity of CAP detection, broaden the linear range of CAP detection, and provide a new choice for CAP detection. This developed sensor was characterized by field emission scanning electron microscopy (FESEM), Fourier transform infrared spectroscopy (FTIR), atomic force microscopic (AFM), cyclic voltammetry (CV), and differential pulse voltammetry (DPV), respectively. Then, the molecular electrostatic potential map (MEP), frontier molecular orbital diagram (FMO), binding energy, selectivity, repeatability, reproducibility, and stability of Arg-MIP/GCE were investigated. In addition, the prepared electrochemical sensor was successfully applied to detect CAP in the actual honey sample.

## 3. Materials and Methods

### 3.1. Reagents

Arginine (Arg, 98%), chloramphenicol (CAP, 98%), potassium chloride (KCl), potassium ferricyanide (K_3_[Fe(CN)_6_]), potassium hexacyanoferrate (II) (K_4_[Fe(CN)_6_]∙3H_2_O), metronidazole (MTR, 99%), fluconazole (FLU, 98%), clarithromycin (CLA, 98%), and tetracycline (TC, 98%) were purchased from Macklin Biochemical Co., Ltd. (Shanghai, China). Sodium phosphate dibasic dodecahydrate (Na_2_HPO_4_·12H_2_O) and Sodium phosphate monobasic dehydrate (NaH_2_PO_4_·2H_2_O) were purchased from Aladdin Chemistry Co., Ltd. (Shanghia, China). All the solutions were prepared with ultrapure water.

### 3.2. Apparatus

All electrochemical investigations were carried out on a CHI760E electrochemical workstation (Chen-Hua, Shanghai, China) with a three-electrode system at room temperature consisting of a silver/silver chloride (Ag/AgCl) electrode, a platinum electrode, and a GCE with a diameter of 4 mm, respectively. The surface morphology and element analysis of the sample were obtained by the instrument of FESEM (FEI nova nanosem 450, Hillsboro, OR, USA), FTIR analysis was executed by Nicolet iS10 Fourier transform infrared spectrometer (Thermo Fisher Scientific Company, Waltham, MA, USA), the microstructure of MIP surface was characterized by AFM (SPA-400 SPM unit, Tokyo, Japan), and the probe type was PPP-SEIHR-20 (SPA-400 SPM unit, Tokyo, Japan).

### 3.3. Computational Studies

The theoretical calculations were performed via the Gaussian 16 suite of programs. The structures of the studied compounds were fully optimized at the B3LYP-D3/6-311+G(d) level of theory. The vibrational frequencies of the optimized structures were carried out at the same level. The structures were characterized as a local energy minimum on the potential energy surface by verifying that all the vibrational frequencies were real. The binding energies (BEs) of the complexes were also calculated to compare the stability of different isomers. The energy of the HOMOs and LUMOs as well as their gaps were calculated at the B3LYP-D3/6-311+G(d) level of theory. The Visual Molecular Dynamics (VMD 1.9.4) program was used to plot the color-filled isosurface graphs to visualize the molecular orbitals and the molecular electrostatic potential (MESP).

### 3.4. Fabrications of MIP and Non-Imprinted Polymers (NIP) Sensor

**Figure 1:**
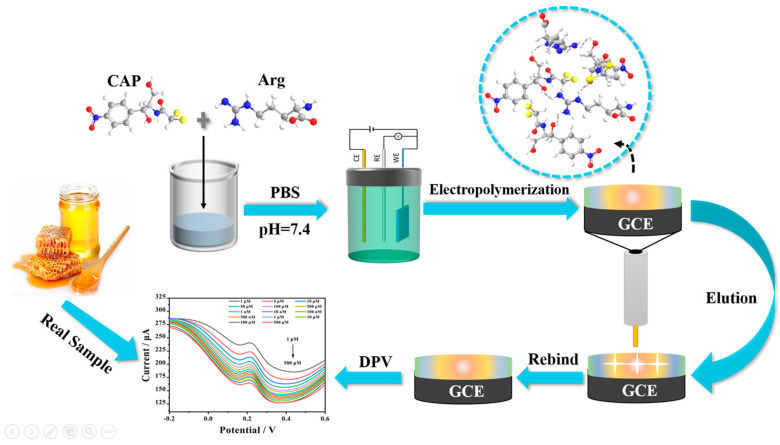
The schematic diagram of the fabrication procedure of the Arg-MIP/GCE sensor.

Before each experiment, it was necessary to use 0.05 μm alumina polishing powder to polish the surface of the GCE on the chamois leather, ultrasonically treat it with ultrapure water and absolute ethanol for 1 min each until the redox peak potential difference of GCE in the CV was less than 110 mV, and then to dry it at room temperature. The pretreated GCE was connected to a three-electrode system and immersed in a phosphate buffer (PBS) (0.1 mol L^−1^, pH = 7.4) electrolyte containing 20 mM Arg and 1 mM CAP. Then, a uniform MIP layer was grown on the surface of the GCE electrode by the CV method in the potential range of −2–2.4 V and scanning speed of 100 mVs^−1^ for 25 cycles, ethanol solution was used to remove the template molecule. The preparation process is shown in Figure 1.

**Figure 2:**
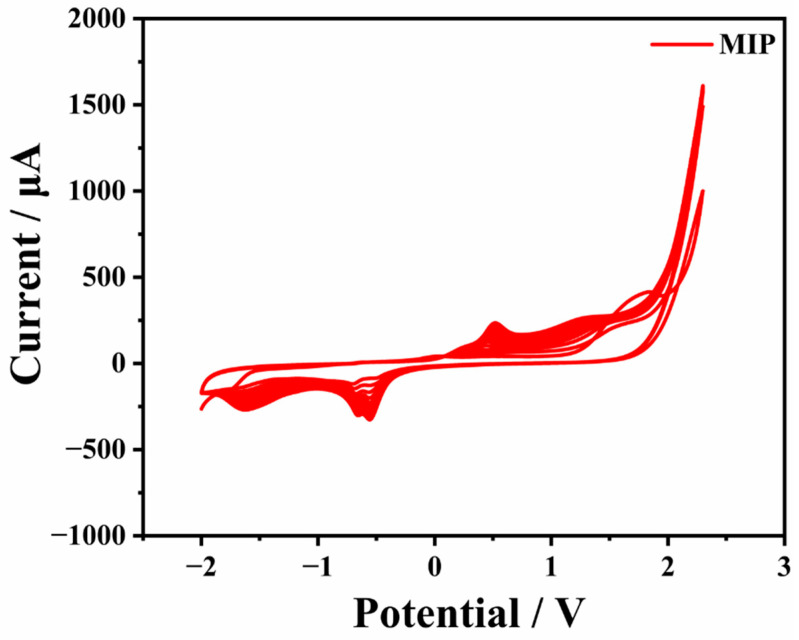
The CV curve of MIP electro-polymerization.

For contradistinction, an Arg-NIP/GCE was produced with the same process mentioned above except without adding CAP in PBS electrolyte. The CV of the electro-polymerization procedure of MIP is displayed in Figure 2.

### 3.5. Electrochemical Measurements

DPV and CV electrochemical experiments were implemented at room temperature using 1.0 mM [Fe(CN)_6_]^3−/4−^ containing 0.1 M KCl as the electrolyte, 100 mV s^−1^ as the scan rate, and the potential range was set at −0.1 to +0.6 V and −0.2 to +0.6 V for CV or DPV measurements, respectively. EIS was performed in the frequency range from 10 kHz to 100 mHz and 5 mV amplitude.

### 3.6. Preparation of Real Samples

In order to test the ability of the prepared sensor for the detection of CAP in real samples, honey was purchased from the local supermarket. A total of 1.0 g of honey was dropped into 30 mL PBS (0.1 mol L^−1^, pH = 7.4) buffer solution, and stirred evenly to make it completely mixed. Then, the different concentrations of CAP solution were dripped into it for detection.

## 4. Results and Discussion

### 4.1. Morphology and Structure of Arg-MIP/GCE

**Figure 3:**
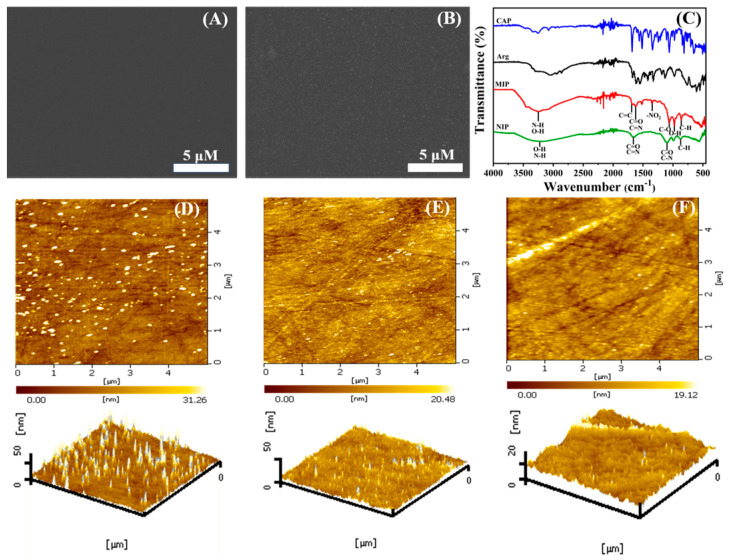
SEM image of (**A**) bare GCE, (**B**) Arg−MIP/GCE, and (**C**) FTIR image of Arg−MIP and Arg−NIP. AFM images of (**D**) Arg−MIP/GCE before elution, (**E**) Arg−MIP/GCE after elution, and (**F**) Arg−NIP/GCE.

The surface morphologies of different samples were characterized by FESEM. From Figure 3A, it can be seen that the bare GCE electrode surface is smooth and flat without foreign matter covering it. When the electro-polymerization occurred, a homogeneous film clearly grew on the bare GCE surface (Figure 3B), which was attributed to the successful formation of MIP by Arg and CAP.

The FTIR spectra of MIP and NIP are shown in Figure 3C. The absorption peak at 867 cm^−1^ is caused by the out-of-plane bending vibration of the C-N group. The peak at 973 cm^−1^ is attributed to the in-plane bending vibration of the O-H group. In addition, the peaks at 1110 cm^−1^ and 1630 cm^−1^ are due to the stretching vibration of the C-O, C-N, C=O, and C=N groups, respectively. The wide absorption band at 3260 cm^−1^ is formed by the stretching vibration of O-H and N-H with intramolecular or free association. These peaks are shared by MIP and NIP, belonging to the common group of CAP and L-Arg. In particular, a pair of absorption peaks at 1350 cm^−1^ and 1510 cm^−1^ in MIP are initiated by asymmetric and symmetric stretching vibrations of the unique -NO_2_ functional group in CAP, and the absorption peak at 1689 cm^−1^ also comes from the stretching vibration of C=C on the benzene ring of the CAP molecule. The existence of these three characteristic peaks also proved the successful synthesis of MIP.

Figure 3D, after the polymerization, there are some relatively uniform spikes on the surface of MIP, and the root mean square (RMS) of roughness is the maximum, with a value of 5.967 nm, which may contribute to the uniform specific sites formed by the combination of CAP and Arg. It also proves the successful preparation of MIP. When CAP molecules are eluted (Figure 3E), the surface is significantly smoother and the surface roughness also decreases to 3.082 nm, which can be explained as the successful elution of CAP molecules. In contrast, NIP (Figure 3F) has the flattest surface and the minimum RMS value, which resulted from the single Arg polymerization.

### 4.2. Computational Studies and Electrochemical Sensing Principle of MIP

**Figure 4:**
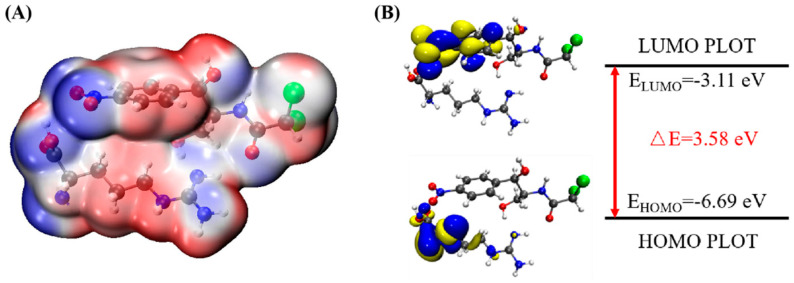
(**A**) MEP maps of Arg−CAP, (**B**) Molecular orbital surfaces and energy levels for HOMO, LUMO, and HOMO−LUMO gap of MIP.

In order to determine the possible binding sites between CAP and Arg, the molecular electrostatic potential (MEP) of MIP was analyzed, and the results of the most stable conformation are shown in Figure 4A. The binding energy is −22.31 kcal·mol^−1^, and other possible configurations are presented in Table 1. In the whole MIP structure, the O and N atoms have higher negative charge distribution and easily accept protons, which are presented as blue regions, while the positive charge distribution of the H atom is relatively high and easily provides protons, which belong to the red region in the MEP. Based on the above results, it can be inferred that the active sites of CAP and Arg are mainly concentrated in the hydrogen atoms of amino, the oxygen atoms of nitro and carbonyl, and the hydrogen atoms and oxygen atoms of hydroxyl. 

Figure 4B is the FMO diagram of the CAP-Arg polymer. The electron transfer occurs between the functional monomer and CAP when the molecular recognition occurs. According to the data in the figure, the HOMO energy of Arg is −6.59 eV, which is higher than that of CAP (−7.69 eV), indicating that Arg is the main electron donor. On the contrary, the LUMO energy of CAP is −3.0 eV, which is lower than that of Arg (−0.44 eV), demonstrating that CAP mainly acts as an electron acceptor. Moreover, compared with CAP (ΔE_gap_ = 4.70 eV) and Arg (ΔE_gap_ = 6.15 eV) monomer, the BPA-Arg complex has a smaller ΔE_gap_ (3.58 eV), which indicates that the BPA-Arg complex forms a more stable complex structure than the monomer through possible receptor-donor interaction.

In summary, it can be seen that during the preparation of Arg-MIP, Arg and CAP mainly bind to each other in the form of hydrogen bonds through functional groups such as amino, nitro, and hydroxyl groups, and a large number of binding sites are generated on the surface of MIP. When the CAP molecule is removed under appropriate elution conditions, many recognition cavities matching the CAP molecule are left on the surface. These cavities are the specific recognition sites in Arg-MIP. The [Fe(CN)_6_]^3−/4−^ probe ions in the electrolyte can reach the electrode surface through these cavities, react, and generate electrical signals. When the eluted MIP sensor is immersed in PBS solution containing different concentrations of CAP, the CAP molecule immediately binds to a specific cavity; this is a recognition relationship similar to a key and lock. When the cavity is filled, the diffusion of [Fe(CN)_6_]^3−/4−^ probe ions in the electrolyte is hindered, resulting in a decrease in the electrical signal. Quantitative detection of CAP can be achieved according to the linear relationship between this concentration and the current decline trend.

### 4.3. Optimization of MIP Fabrication

The electrochemical performance of MIP is related to many preparation factors. Therefore, in order to obtain the MIP electrochemical sensor which has the best detection performance for CAP, the parameters such as the number of electro-polymerization cycles, molar ratio of functional monomer and template molecule, pH value of electrolyte, elution time, and incubation time were experimentally optimized before the preparation process. The best preparation condition was selected by comparing the difference of DPV current before and after incubation of CAP. Each group of experiments was repeated three times (n = 3) and the average value was taken for plotting.

**Figure 5:**
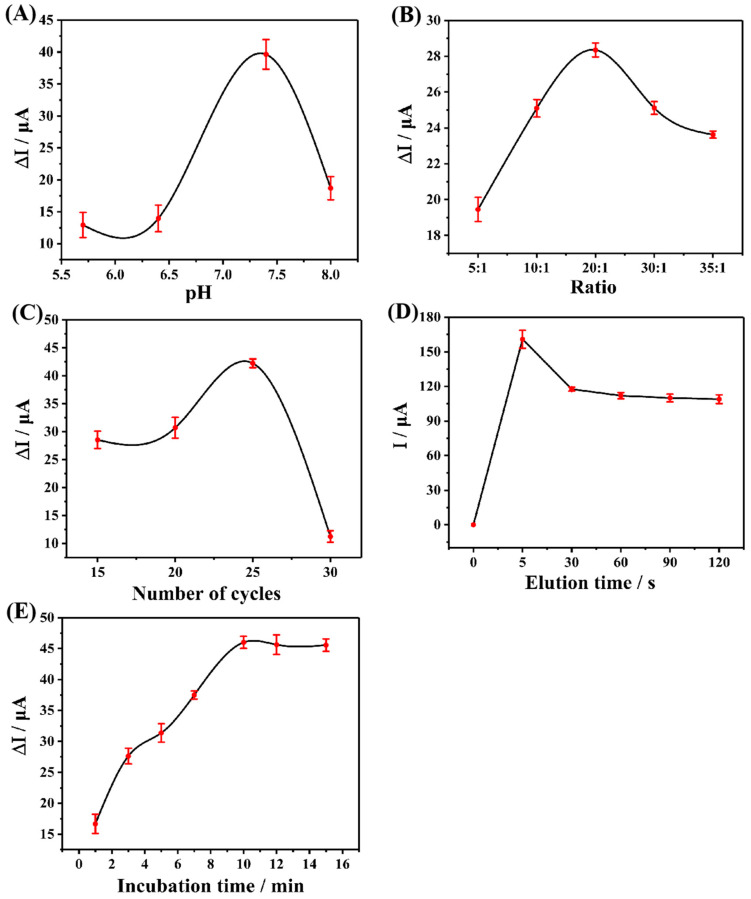
(**A**) Effect of pH Value of electrolyte during the preparation process, (**B**) effect of different ratios of the functional monomer to template molecules, (**C**) effect of the electro-polymerization cycles during the preparation process, (**D**) effect of elution time on the current response of the sensor to CAP, and (**E**) effect of incubation time on the current response of the sensor to CAP.

The pH value of the electrolyte will have a certain impact on the electro-polymerization process, the stability of CAP and Arg molecules, and their interaction, so the optimization of the pH value of the electrolyte cannot be ignored. PBS buffer solution with pH values from 5.7 to 8.0 was selected as the electrolyte for the test. The results are shown in Figure 5A. With the increase of pH value, the DPV current difference before and after incubation increased, reaching the highest point at 7.4 and then showing a downward trend, indicating that the electrolyte with a pH value of 7.4 could produce the most effective active sites of CAP during the electro-polymerization process, and get the largest response value. Hence, the preparation pH value used in the subsequent experiment was 7.4.

The molar ratio of functional monomer to template molecule is directly related to the number of active recognition sites in the final MIP. Therefore, to obtain MIP with excellent performance, the molar ratio must be optimized. Figure 5B reveals the relationship between the current difference before and after the incubation of CAP and the molar ratio. It can be seen that before 20:1, with the increase of the molar amount of functional monomers, the quantity of recognition sites in the prepared MIP increases accordingly, and the adsorption capacity of CAP molecules enhances continuously, reaching the maximum at 20:1. After that, due to the increasing number of functional monomers, the MIP is cross-linked too much and the recognition site is covered, which makes it difficult for CAP molecules to reach the recognition site. Therefore, the adsorption capacity decreases gradually, and the DPV current difference shows a downward trend.

The thickness of MIP is also an important factor affecting its final electrochemical performance. In the preparation process, the thickness of MIP is adjusted by adjusting the number of cycles of electro-polymerization. The results are shown in Figure 5C. Between 15 and 25 cycles, the DPV current difference also shows an upward trend with the increase in the number of cycles. This indicates that the increasing number of cycles before 25 cycles promotes the formation of MIP, which forms sufficient active recognition sites to fully recognize CAP molecules, and the excessive cycle after 25 will lead to excessive MIP thickness, which increases the electron transfer resistance and elution difficulty. As a result, the final MIP activity recognition sites are not fully exposed and the performance is reduced.

After the preparation of MIP, CAP molecules need to be removed from the MIP surface by elution, leaving specific recognition sites. As a result, it is desirable to select the most suitable elution time to expose the most recognition sites without damaging the MIP structure. In this study, taking into account that CAP and Arg are mainly combined by hydrogen bonds, ethanol solution was selected as an eluent. Figure 5D demonstrates that the current value almost no longer changes with the increase of elution time after 30 s, which indicates that the recognition site has been fully exposed after 30 s.

The incubation time of eluted MIP was optimized in PBS solution containing 5 μM CAP. It can be seen from Figure 5E that the current difference tended to be stable after 10 min, indicating that the recognition sites on MIP reached saturation after incubation for 10 min. Therefore, 10 min was selected as the optimal adsorption time.

### 4.4. Electrochemical Characterization

**Figure 6:**
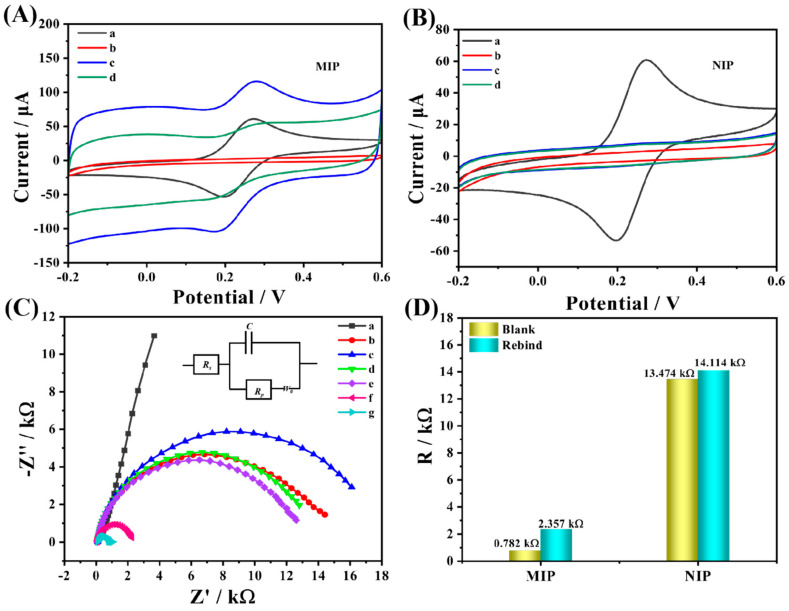
(**A**) CV of Arg−MIP and (**B**) Arg−NIP in 1 Mm [Fe(CN)_6_] ^3−/4−-^ solution containing 0.1 M KCl: (a) bare GCE, (b) before CAP elution, (c) after CAP elution, and (d) after removal of template incubated in CAP solution for 10 min. (**C**) EIS of different types of modified electrodes in 1.0 mM [Fe(CN)_6_] ^3−/4−^ solution contains 0.1 M KCI for (a) bare GCE, (b) Arg−NIP, (c) Arg−MIP, (e) after the elution of the CAP from NIP and (g) MIP, (d) after CAP rebind of NIP, and (f) MIP. (**D**) Resistance changes of MIP and NIP before and after CAP adsorption.

In order to investigate the electrochemical performance of the prepared sensor, CV tests were performed on different modified electrodes using 0.1 M KCl solution containing 1.0 mM [Fe(CN)_6_] ^3−/4−^ as the electrolyte in the potential range of −0.2–0.6 V. As shown in Figure 6A, a pair of obvious redox peaks existed on the bare GCE electrode. When the dense and non-conductive MIP film was attached to the GCE surface by means of electro-polymerization, which hindered the electron transfer, both redox peaks disappeared, proving the successful preparation of MIP. Afterward, as the non-conductive CAP molecules were eluted, the electrode surface exposed certain recognition cavities, which allowed the probe ions to pass smoothly. Simultaneously, the redox response current was significantly enhanced after elution, compared with GCE, since the presence of Arg promoted the electrochemical performance. After the CAP molecule was reabsorbed on the recognition sites on the MIP surface, the electrical signal showed a significant decrease compared with that before adsorption, which was caused by the specific recognition response of the MIP to the CAP molecule, compared with the NIP (Figure 6B) where no obvious current signal difference was observed before and after the adsorption of the CAP molecule due to the absence of the specific recognition sites of the CAP present. 

To further investigate the electron transfer changes of the different modified electrodes, EIS tests were conducted, and the results were consistent with the previous CVs, as demonstrated in Figure 6C. When MIP polymerized on the surface of the GCE, electrode R_ct_ increased remarkably, proving that the electron transfer was severely hindered by the non-conducting molecularly imprinted film; similarly, the electron transfer resistance was enhanced after NIP polymerization. However, due to the lack of non-conducting molecule CAP, its R_ct_ value was smaller than that of MIP. After the elution procedure of MIP, specific recognition cavities matching the shape of CAP molecule appeared on its non-conductive surface, and the electron transfer resistance was reduced. When some cavities were filled again after adsorption, R_ct_ increased. The comparison of NIP and MIP after the same operation is illustrated in Figure 6D. The R_ct_ value of NIP after elution was significantly higher than that of MIP, which was due to the absence of CAP. On the contrary, the impedance value of MIP was significantly smaller after elution owing to the presence of CAP molecules, and the change of impedance value was about 1575 Ω after resorption occurred, which could be attributed to the existence of a large number of CAP-specific recognition sites in the structure of MIP, while the impedance value of NIP was significantly lower than that of MIP because of the absence of specific recognition sites. As a result, the change in impedance was significantly lower than that of the MIP.

**Figure 7:**
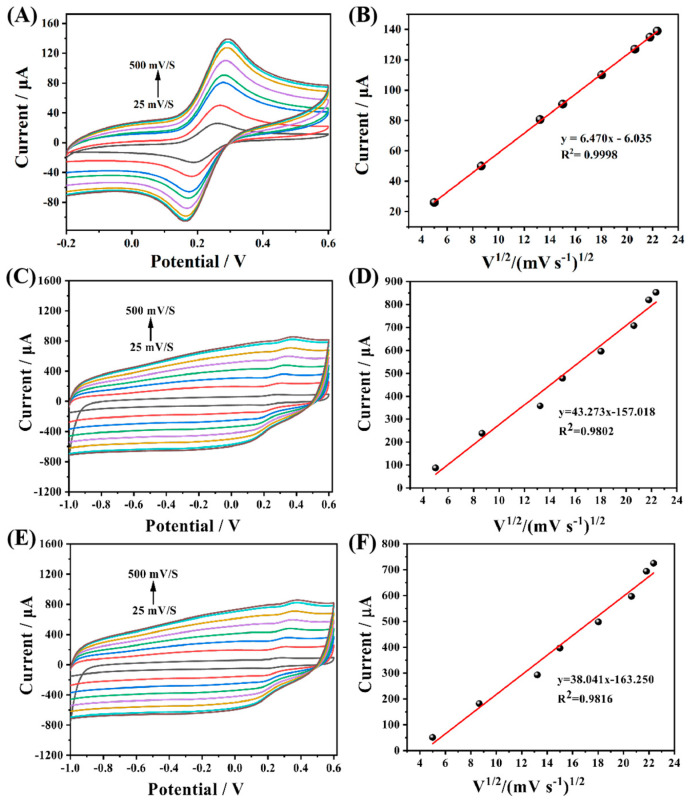
CVs with scan rates of 25 to 500 mV·s^−1^ of (**A**) GCE, (**C**) Arg−MIP/GCE after elution, and (**E**) Arg−MIP/GCE after incubation. The effective area of (**B**) GCE, (**D**) Arg−MIP/GCE after elution, and (**F**) Arg−MIP/GCE after incubation.

Subsequently, CV tests were carried out on GCE and MIP template molecules before and after elution at different scanning rates. The test results showed that the redox peak current value had a good linear relationship with the square root of scanning speed, which proved that the reaction on the electrode surface was mainly controlled by diffusion. The active surface area of the different modified electrodes can be calculated according to the Randles–Sevcik equation as follow:

Ip = (2.69 × 10^5^) n^3/2^AD^1/2^cv^1/2^

where n is the number of electron transfers during the experiment (n = 1), A represents the effective surface area of different electrodes, D is the diffusion coefficient (D = 7.6 × 10^−6^ cm^2^ s^−1^), c is the concentration of the basal solution, and v is the scan rate of CV. The experimental results are linearly fitted with the oxidation peak current as the vertical coordinate and v^1/2^ as the horizontal coordinate, and the active area (A) can be calculated by the slope. Based on this method, a linear fit to the three different electrodes demonstrates that their electrochemically active areas are 0.100 cm^2^ (Figure 7B), 0.670 cm^2^ (Figure 7D) and 0.587 cm^2^ (Figure 7F), respectively. The results indicate that the active specific surface area of MIP after elution has increased nearly 7-fold compared with that of GCE, and the active specific surface area also decreased significantly after resorption, further verifying the existence of a large number of active cavities on MIP and the specific adsorption recognition of CAP molecules.

### 4.5. Arg-MIP/GCE Electrochemical Detection Performance and Mechanism

In the process of MIP electro-polymerization preparation, Arg and CAP are combined in the form of hydrogen bonds through functional groups such as amino and hydroxyl groups, which generates a large number of binding sites on the polymer surface. When the CAP molecules are removed under suitable elution conditions, the surface is left with many recognition cavities that match with the CAP molecules, so that the probe ions in the electrolyte can reach the electrode surface through the cavities to trigger an electrical signal. When the eluted MIP sensor is immersed in PBS solution containing different concentrations of CAP, the CAP molecules immediately combine with the specific cavities, similar to a key-lock recognition relationship. After the cavities are filled, the diffusion of probe ions in the electrolyte is hindered, resulting in a decrease in the electrical signal, then the CAP can be quantitatively tested.

**Figure 8:**
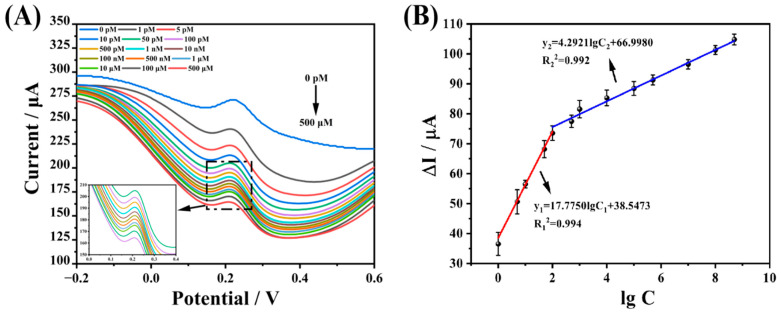
(**A**) DPV of Arg-MIP/GCE sensor after incubation in different concentrations of CAP. (**B**) The linear relationship between ∆I and logarithm of concentration of Arg-MIP/GCE and Arg-NIP/GCE.

In this study, the linear range of CAP was detected using DPV as an electrical signal detection technique, with 0.1 M KCl solution containing 1.0 mM [Fe(CN)_6_]^3-/4-^ as the probe solution, as shown in Figure 8A. The current response of DPV tends to decrease as the CAP concentration increases, using the difference between the current decrease before and after the logarithm of the concentration to do a linear fit, and the fitting results are displayed in Figure 8B. The linear response of the prepared Arg-MIP/GCE electrochemical sensor to CAP ranges from 1 × 10^−12^ mol L^−1^ to 5 × 10^−4^ mol L^−1^, which can be divided into two segments; the linear equations are expressed as ∆I = 17.775 lgC + 38.547 (R^2^ = 0.994), ∆I = 4.217 lgC + 67.539 (R^2^ = 0.993), respectively. The LOD is 1.36 × 10^−13^ mol L^−1^ (LOD = 3 × SDb/m), and the LOQ (LOQ = 10 × SDb/m) is 4.53 × 10^−13^ mol L^−1^.

Compared with other previous studies (Table 2), in this study it is clear that the Arg-MIP/GCE electrochemical sensor not only has a wider linear detection range of CAP without modifying other nanomaterials, but also has a lower detection limit and higher sensitivity, which can achieve the high-performance detection of CAP molecules with low cost, thus giving this method more advantages and broader application prospects.

### 4.6. Selectivity and Interference Tests of the Arg-MIP/GCE Sensor

**Figure 9:**
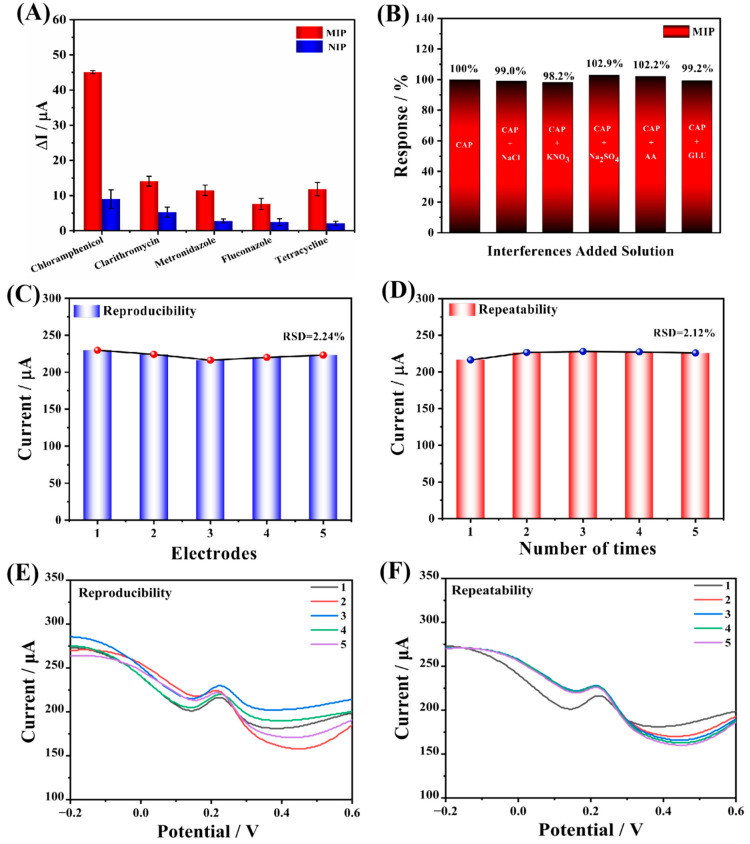
(**A**) DPV current response difference of Arg-MIP/GCE and Arg-NIP/GCE for different antibiotics. (**B**) The anti-interference performance of Arg-MIP/GCE sensors. The reproducibility (**C**) and repeatability (**D**) of Arg-MIP/GCE. The row DPV curves for reproducibility (**E**) and repeatability (**F**) of Arg-MIP/GCE.

Selectivity and anti-interference are two important parameters used to evaluate the performance of electrochemical sensors. In this study, the DPV method was used to test them, and several commonly used veterinary antibiotics (clarithromycin, metronidazole, fluconazole, tetracycline hydrochloride) were chosen for the selectivity test. The prepared Arg-MIP/GCE sensor was immersed in 5 μM of veterinary antibiotics and the results of MIP and NIP were compared, as shown in Figure 9A. There, it can be seen that the Arg-MIP/GCE sensor has the highest response current difference to CAP, and the imprinting factor (IF) is 5.007, which is nearly four times the response value of other antibiotics. The response current values of the Arg-MIP/GCE sensor at 5 μM are compared with other veterinary antibiotics and calculated; the selectivity coefficients of CAP over clarithromycin, metronidazole, fluconazole, and tetracycline hydrochloride were determined to be 3.196, 3.919, 5.904, and 3.809 respectively. This proves that the prepared sensor has good selectivity, and the response difference of NIP is significantly lower than that of MIP, which can be attributed to the lack of specific recognition sites for CAP molecules on NIP. In addition, the Arg-MIP/GCE sensor has no obvious change in the detection current of CAP when we add other interference substances such as glucose, ascorbic acid, Na^+^, and K^+^ with the content of CAP 100 times (Figure 9B), indicating that the sensor has ideal anti-interference. In conclusion, good selectivity and anti-interference lay the foundation for the application of Arg-MIP/GCE sensor in a real detection environment. 

### 4.7. Reproducibility, Repeatability, and Stability

**Figure 10:**
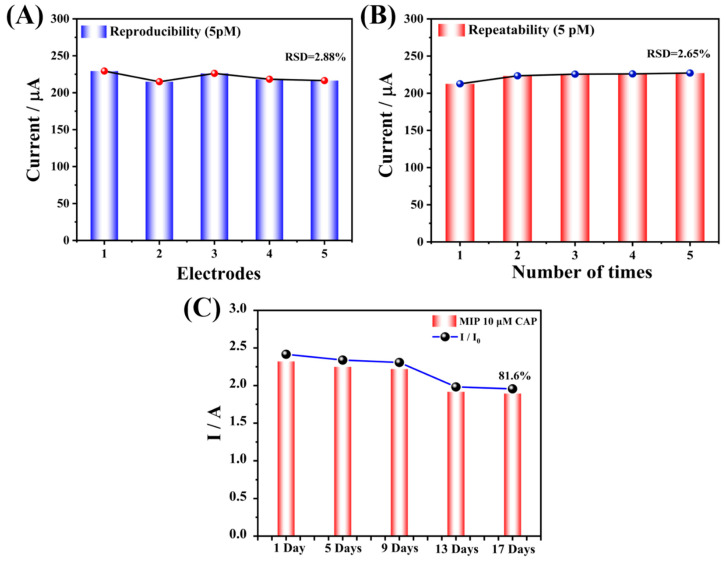
(**A**) The reproducibility and (**B**) repeatability of Arg-MIP/GCE at low concentration (5 nM). (**C**) The stability of the Arg-MIP/GCE sensor.

In order to research the reproducibility and repeatability of the prepared Arg-MIP/GCE sensor, five identical electrodes were prepared to detect CAP at the same concentration (5 μM). As is depicted in Figure 9C, the current value changed little, and the relative standard deviation (RSD) was 2.24%. At the same time, the same electrode was used to repeatedly test CAP at the same concentration five times (Figure 9D), and the test results also did not show significant changes (RSD = 2.12%). At the same time, the repeatability and reproducibility of the sensor at low concentration (5 pM) were also tested. The results are shown in Figure 10. These results reveal that the prepared sensor had reliable reproducibility and repeatability. Subsequently, the prepared electrode was stored at 4 °C and measured every four days. After 17 days, the measured response value remained 81.6% of the original response value (Figure 10C), indicating that it had acceptable stability.

### 4.8. Real Sample Analysis

Since chloramphenicol has been banned from food animals, of its presence in honey has been repeatedly reported. It is of great safety significance to monitor CAP in honey. Therefore, honey was selected as a real sample for quantitative detection of CAP (Table 3). Different concentrations of CAP (5 pM, 10 pM, 100 pM) were added to honey samples diluted with PBS, and the average value was removed after three iterations of each test. After calculation, the CAP detection recovery of the Arg-MIP/GCE sensor was 91.36−104.6%. RSD values were all less than 5%. The results show that the Arg-MIP/GCE sensor has the ability to detect CAP in real samples, even at very low concentrations.

## 5. Conclusions

In this work, we developed a MIP electrochemical sensor with simple preparation, low cost, and no modification of nanomaterials to complete the high-performance detection of CAP by using arginine as functional monomer. The prepared MIP electrochemical sensor has a wide linear range for CAP detection from 1 × 10^−12^ mol L^−1^ to 5 × 10^−4^ mol L^−1^, and the detection limit is as low as 1.36 × 10^−13^ mol L^−1^, which can achieve the monitoring of very low concentration CAP. In addition, the Arg-MIP/GCE sensor has excellent selectivity, anti-interference, repeatability, and reproducibility, and can also complete the low concentration CAP detection in real honey samples. Based on the trend of portability and simplification of the electrochemical sensor in the future, the Arg-MIP/GCE sensor not only reduces the production cost and simplifies the production process, but also improves the detection performance for CAP. It provides a new choice for the preparation and optimization of new sensors, has practical application value and application prospect.

## Figures and Tables

**Table 1:** ESP diagrams and binding energies of other CAP-Arg configurations.

Geometry	ESP
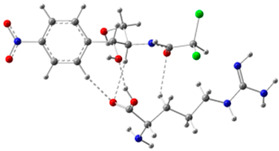	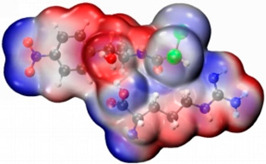
**BE = −20.30 kcal·mol^−1^**	
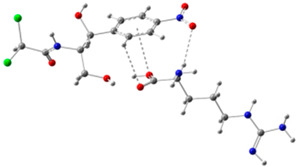	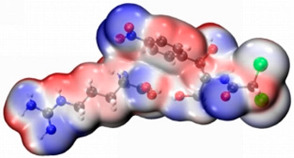
**BE = −19.78 kcal·mol^−1^**	
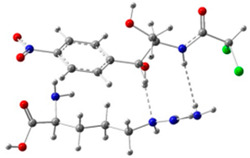	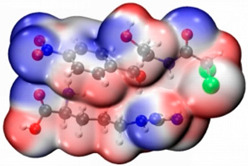
**BE= −18.51 kcal·mol^−1^**	

**Table 2:** Comparison of the developed method with several methods for CAP detection.

Sensor	Method	Linear Range/μM	LOD/μM	Reference
MoS_2_/PANI/CPE	DPV	0.1–100	0.069	[30]
MoS_2_/2D rGO/GCE	DPV	5–35	1	[31]
PCN-222-CHIT/PEDOT/ITO	DPV	0.01–0.8	0.0018	[1]
rGO/PdNPs/GCE	DPV	0.05–1	0.05	[6]
3D CNTs/Cu NPs/MIP	CV	10–500	10	[32]
g-C_3_N_4_/MnWO4/GCE	DPV	4 × 10^−3^–7.1 × 10^−2^	1.03 × 10^−3^	[16]
3-ampy-RGO/Ag NPs/MIP/GCE	EIS	1 × 10^−6^–1 × 10^−3^	0.3 × 10^−6^	[2]
Fe_3_O_4_/GCE	SWV	0.09–47	0.09	[33]
MOF/MoS_2_	Fluorescence	0–0.3	0.2 × 10^−3^	[34]
Bi_2_WO_6_	photoelectrochemical	0.5 × 10^−4^–0.1	1.2 × 10^−5^	[35]
MIP/Pt TFMEs	SWV	9 × 10−4–1 × 10−2	0.39 × 10−3	[36]
MWCNTs@MIP/CKM-3/P-r-GO/GCE	DPV	5 × 10^−3^–4	1 × 10^−4^	[5]
COF-AI/Co3O4/MIP/Au	ECL	5 × 10^−7^–4 × 10^−4^	1.17 × 10^−7^	[37]
Arg-MIP/GCE	DPV	1 × 10^−6^–500	1.36 × 10^−7^	**This work**

**Table 3:** Determination of CAP in honey samples using Arg-MIP/GCE sensor.

Sample	Add (pM)	Found (pM)	Recovery (%)	RSD (%) (n = 3)
Honey1	5	5.1	102	2.52
Honey2	10	10.46	104.6	0.61
Honey3	100	91.36	91.36	4.83

## Data Availability

Not applicable.
